# Supplementary material for: Ripplocations provide a new mechanism for the deformation of phyllosilicates in the lithosphere
Source: Nat Commun. 2019 Feb 15;10:686. doi: 10.1038/s41467-019-08587-2 (PMC6377708; doi:10.1038/s41467-019-08587-2)
Supplement: Supplementary file 1 — Supplementary Information [file 41467_2019_8587_MOESM1_ESM.pdf]

Supplementary Information for:

Ripplocations provide a new mechanism for the deformation of phyllosilicates in the lithosphere

Aslin et al.

Supplementary Note 1: Kink band morphology and formation

As described in the main text of this article, previous studies of kink bands have attributed their formation to the development of complex arrays of dislocation walls, which result in bending of the (001) plane within a finite region on the order of 1-2 μm[1,2]. However such a mechanism is unable to account for the degree of lattice curvature that we report in this study. In addition basal dislocations alone struggle to explain observed kink band asymmetry. Etheridge et al.[1] lay out a detailed description of kink band morphology and constitution. They define kink band asymmetry as:

$$|\phi - \phi_K|$$

Where ϕ is the angle between (001) in the limb outside the kink band and the axial plane of the KBB and ϕ_K is the angle between (001) in the limb inside the kink band and the axial plane of the KBB. The angle between the basal planes of the undeformed limb of the kink band and the deformed limb is known as the angle of bending (ω). According to Etheridge et al.[1] kink asymmetry is dependent on temperature and orientation with greater amounts of asymmetry in specimens deformed along [010] and in those deformed at higher temperatures, however, asymmetric kink bands occur at all conditions. This asymmetry of kink bands is the most difficult aspect of their morphology to explain through basal slip alone as it, by necessity, results in an expansion of the basal plane spacing in the region contained within the kink band. Previous authors[1–3] agree that this asymmetry, and the resultant c-axis parallel strain, requires some other deformation mechanism in addition to basal dislocation glide, and invoke non-basal slip and/or fracturing to account for this, despite little unequivocal evidence. Ripplocations as a recently recognised defect[4] are directly characterised by such an expansion in the basal plane spacing and, consequently, perfectly describe this phenomenon. Descriptions of kink bands on the scale of both optical microscopy[1] and TEM[2] report basal delaminations around and across KBBs. These delaminations possess variable symmetry and curvature and are similar in form to those described in this study and associated with the release of stored c-axis parallel strain. The distribution of these structures along KBBs is also in strong

agreement with the computational models of Gruber et al.[5] (supplementary movie 4 of Gruber et al.[5]) which model kink band formation using ripplocations instead of basal dislocations.

Supplementary Note 2: Sample locations and geological background

The three naturally deformed samples used in this study were collected from viscously deformed granitic orthogneiss rocks of the Cossatto-Mergozzo-Brissago (CMB) line and the associated Pogallo line shear zones in north western Italy. The CMB line is a vertical tectonic discontinuity which separates the lower crustal Ivrea-Verbano zone to the NW from the mid-upper crustal Serie dei Laghi to the SE. It was active at lower amphibolite facies conditions during the Permian and accounts for several tens of km of lateral displacement[6]. The Pogallo line is a slightly younger amphibolite to upper greenschist facies shear zone which cuts the CMB line at a very low angle[7]. The orthogneiss samples used were composed primarily of quartz and both plagioclase feldspar and K-feldspar. Micas constituted between 15% and 20% of the samples with biotite usually comprising just over half of this with the remainder being muscovite.

The undeformed sample was a block of Westerly granite from Rhode Island, USA, which is a medium grained, relatively biotite-rich (5-10%) intrusive igneous rock. It is a useful standard material used extensively for deformation experiments in rock mechanics as it is homogeneous and has undergone very limited natural deformation.

Supplementary Note 3: Brittle v viscous character of ripplocations and the pressure sensitivity of micas

It is not clear whether deformation by ripplocations falls under the category of a brittle or a viscous mechanism. Unlike a brittle fracture they do not represent the permanent breaking of bonds but nor do they occur through in plane motion of one plane of atoms relative to another as in dislocation glide. In addition, their effects, such as kinking, are prevalent in phyllosilicates across the entire range of conditions within the Earth's crust and occur alongside both classically brittle and viscous

processes. A key effect of ripplocations is the expansion of the unit cell parallel to the c-axis. Where this occurs in a recoverable manner, which essentially consist of stretching the interlayer bonding, it may be that this can be considered a viscous mechanism. However, once the unit cell expansion is too great, and the bonding is broken rather than stretched, a true delamination is formed, and the mechanism is better described as brittle. In reality, the boundary between these two states may be somewhat blurred, micro-scale delaminations may exist in a transient form that are small enough to heal and leave behind a regular lattice.

Viscous dislocation glide is possible in micas even at the pressure and temperature conditions of the Earth's surface[8], where brittle mechanisms dominate in most minerals. Brittle behaviour is characterised by a proportional increase in yield strength with increasing lithostatic pressure, as higher pressures increase the energy required to break bonds. This response is observable in mica rich rocks up to their dehydration temperature[9] but is often coupled with a lack of microstructural evidence for brittle deformation. This unusual pressure sensitivity of phyllosilicates can now be explained by ripplocations. Gruber et al.[5] showed that the nucleation and motion of ripplocations is strongly dependent on their confinement (or the lithostatic pressure). At the same time, they do not result in fractures or other brittle features but rather produce a bending of lattice planes and kinking. Such observations strongly suggest that ripplocations are the mechanism responsible for this, until now, poorly understood mechanical response of phyllosilicates.

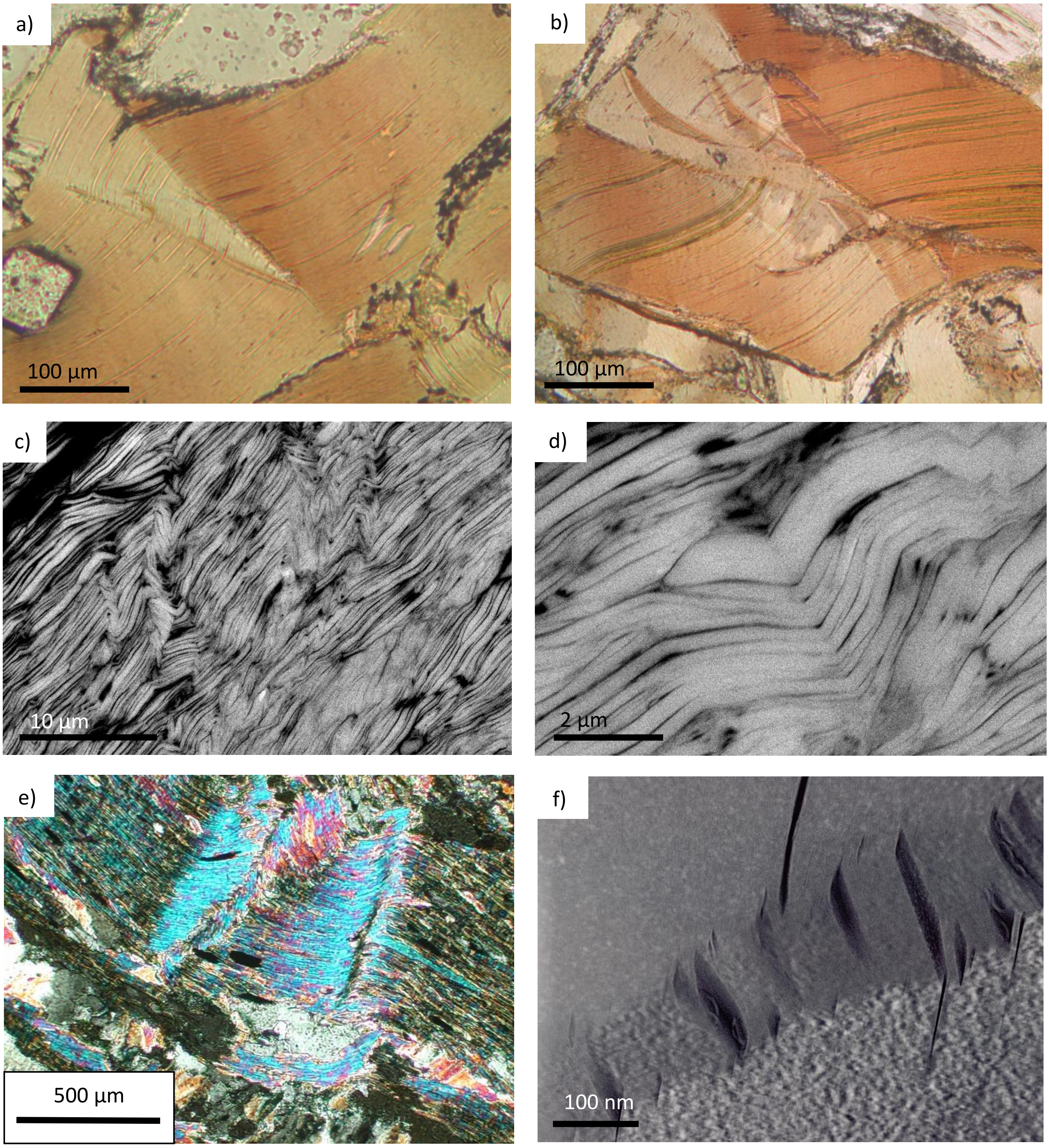

Supplementary Figure 1. Examples of various forms of kinking and folding in micas from different conditions and at a range of scales demonstrating its importance as a ubiquitous process of deformation in phyllosilicates. (**a**) and (**b**) Optical micrographs (plane polarized light) of biotite from sample CMB5A, an amphibolite facies mylonite from the Cossatto-Mergozzo-Brisaggo line used in this study. Note how the sharpness of individual kink band boundaries often varies along their length with sharp kinks sometimes grading into more gentle bends. (**a**) A simple single kink band which serves to locally increase the width of the grain and decrease its length. The two (possibly three) kink band boundaries either side of the kink band meet and annihilate towards the bottom of the grain. (**b**) A more complex network of kink bands which facilitates greater basal plane parallel compression and c-axis parallel expansion of the grain. (**c**) and (**d**) Back scattered electron images of muscovite deformed experimentally under conditions of simple shear (**c**) and torsion (**d**). Kinks are abundant and accompanied by basal layer delaminations. In simple shear (**c**) the kinks show a vergence towards the direction of shear. Scale bars are (**e**) Optical micrograph (crossed polarized light) of kinked metamorphic muscovite. (**f**) TEM micrograph of a simple kink band in biotite from sample POG 7 Showing that kinking occurs even at the nano-scale and demonstrating that where kink band asymmetry is substantial, widening of the basal spacing or delamination is geometrically necessary. (**c**) and (**d**) are from Mariani (2002)[10], (**e**) courtesy of Dr Giles Droop.

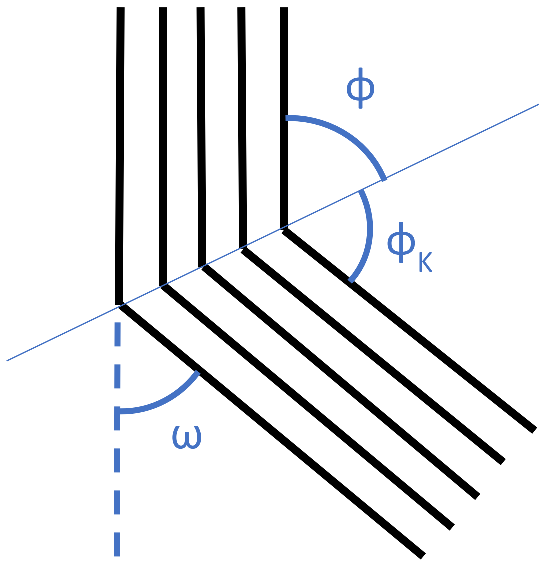

Supplementary Figure 2. Kink band morphology. Designation of angles within a kink band boundary as defined by Etheridge et al.[16]. ω is the angle of bending, ϕ is the angle between (001) in the limb outside the kink band and the axial plane of the KBB and $ϕ_K$ is the angle between (001) in the limb inside the kink band and the axial plane of the KBB.

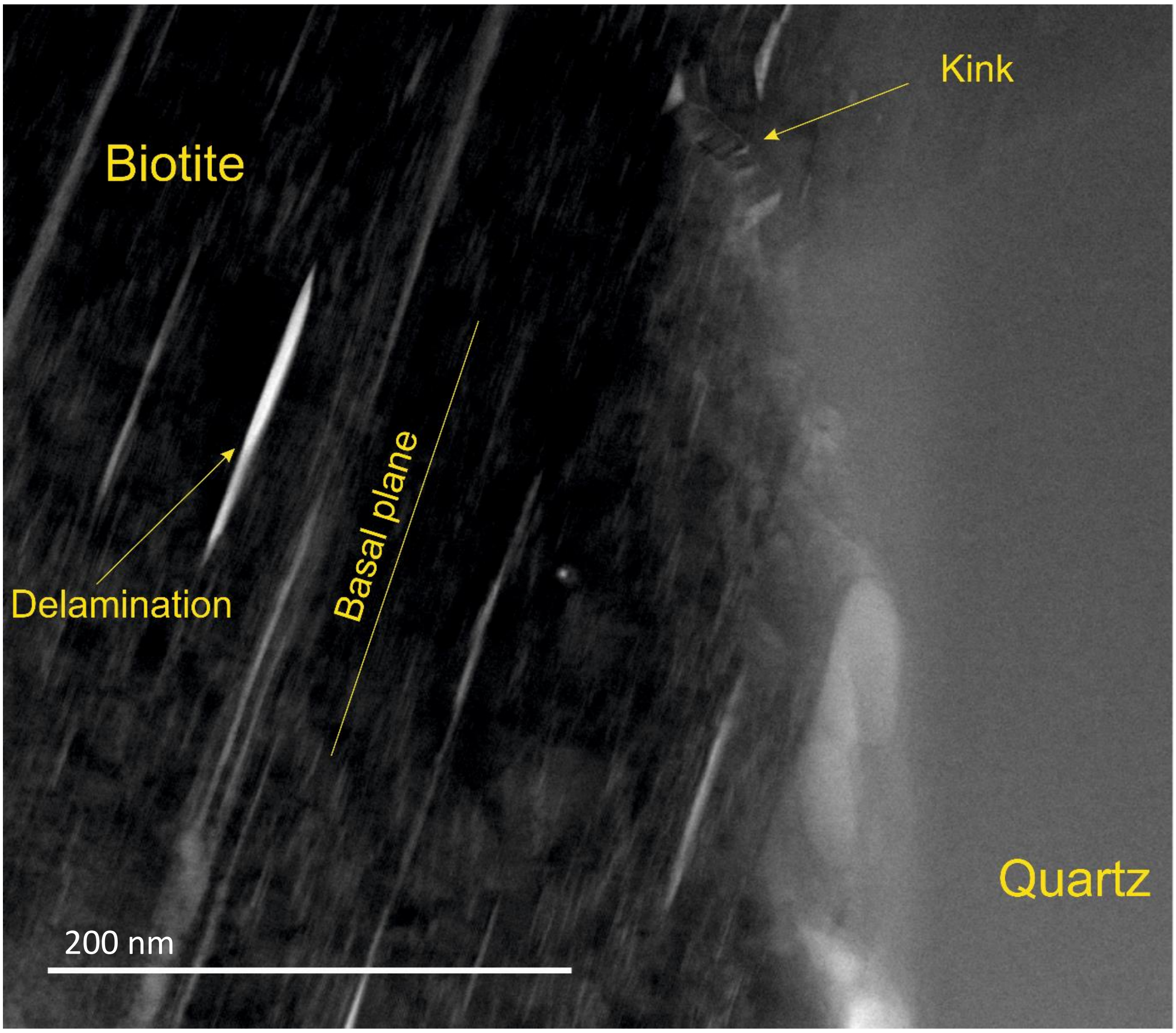

Supplementary Figure 3. Brightfield TEM image from sample CMB5A prepared by FIB, showing delamination and expansion structures of variable scales within biotite as well as a nanometer-scale kink band (indicated in the upper part of the image) which may be related to local differential stresses associated with the proximity of a biotite-quartz phase boundary.

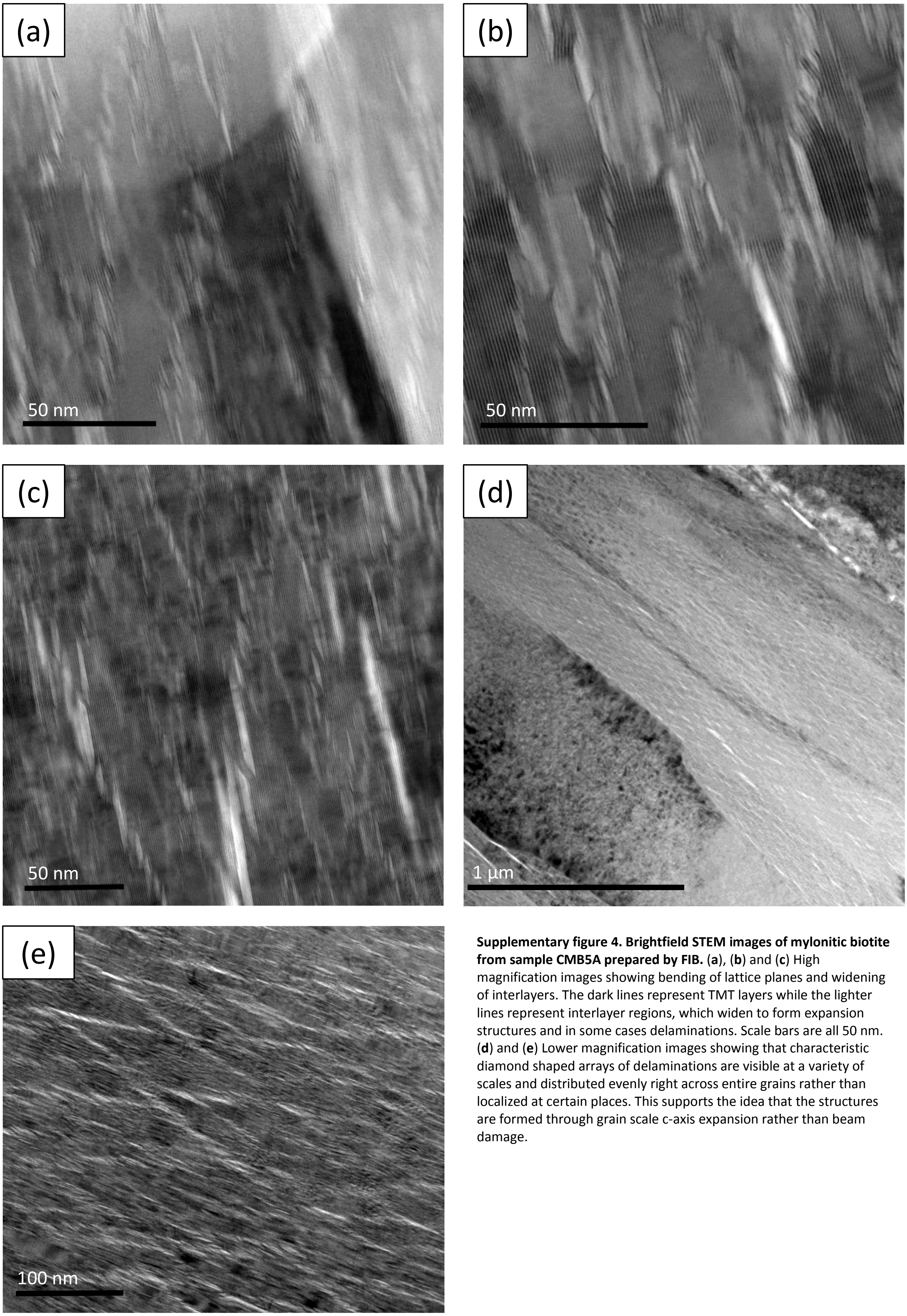

Supplementary figure 4. Brightfield STEM images of mylonitic biotite from sample CMB5A prepared by FIB. (**a**), (**b**) and (**c**) High magnification images showing bending of lattice planes and widening of interlayers. The dark lines represent TMT layers while the lighter lines represent interlayer regions, which widen to form expansion structures and in some cases delaminations. Scale bars are all 50 nm. (**d**) and (**e**) Lower magnification images showing that characteristic diamond shaped arrays of delaminations are visible at a variety of scales and distributed evenly right across entire grains rather than localized at certain places. This supports the idea that the structures are formed through grain scale c-axis expansion rather than beam damage.

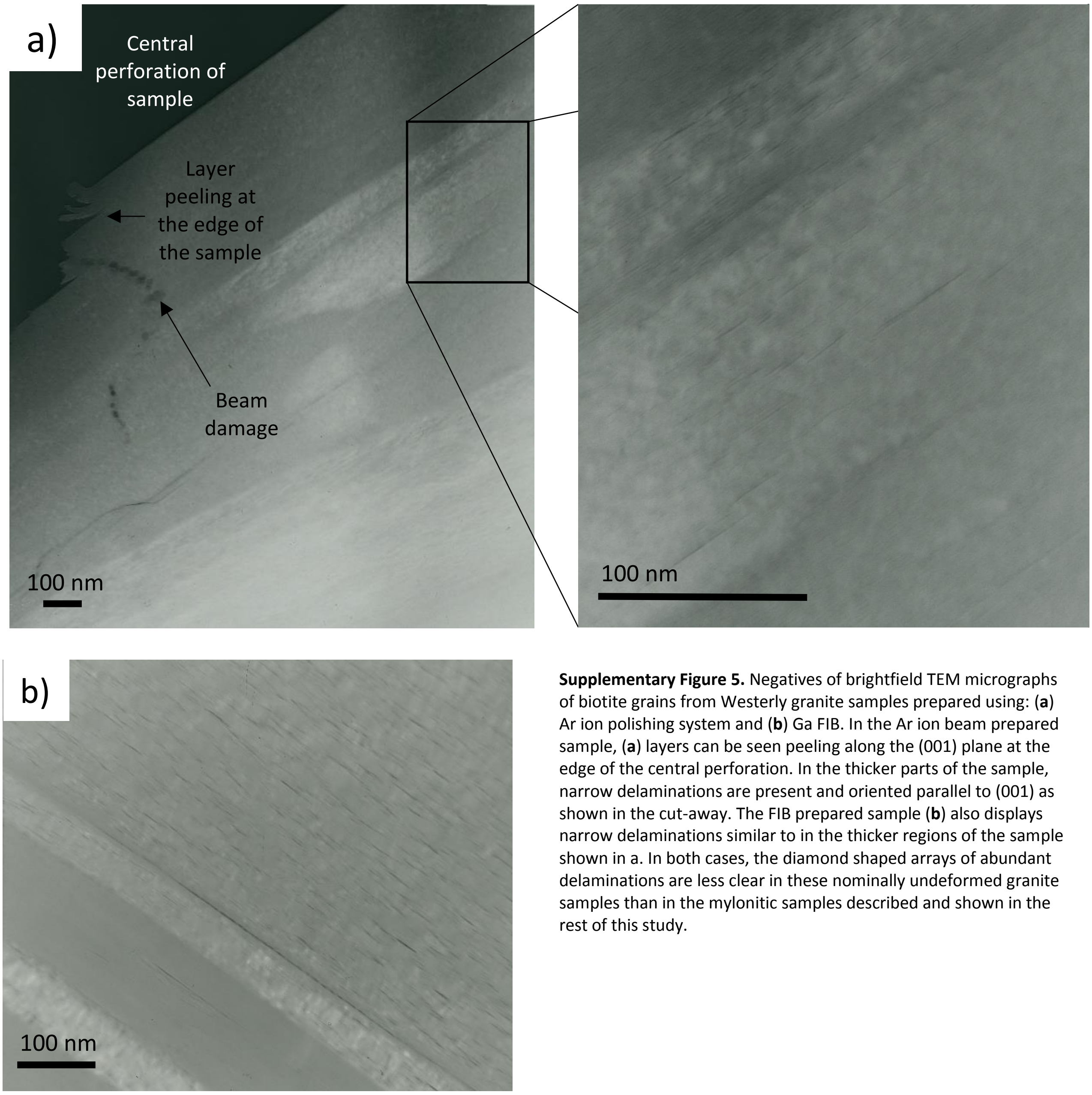

Supplementary Figure 5. Negatives of brightfield TEM micrographs of biotite grains from Westerly granite samples prepared using: (**a**) Ar ion polishing system and (**b**) Ga FIB. In the Ar ion beam prepared sample, (**a**) layers can be seen peeling along the (001) plane at the edge of the central perforation. In the thicker parts of the sample, narrow delaminations are present and oriented parallel to (001) as shown in the cut-away. The FIB prepared sample (**b**) also displays narrow delaminations similar to in the thicker regions of the sample shown in a. In both cases, the diamond shaped arrays of abundant delaminations are less clear in these nominally undeformed granite samples than in the mylonitic samples described and shown in the rest of this study.

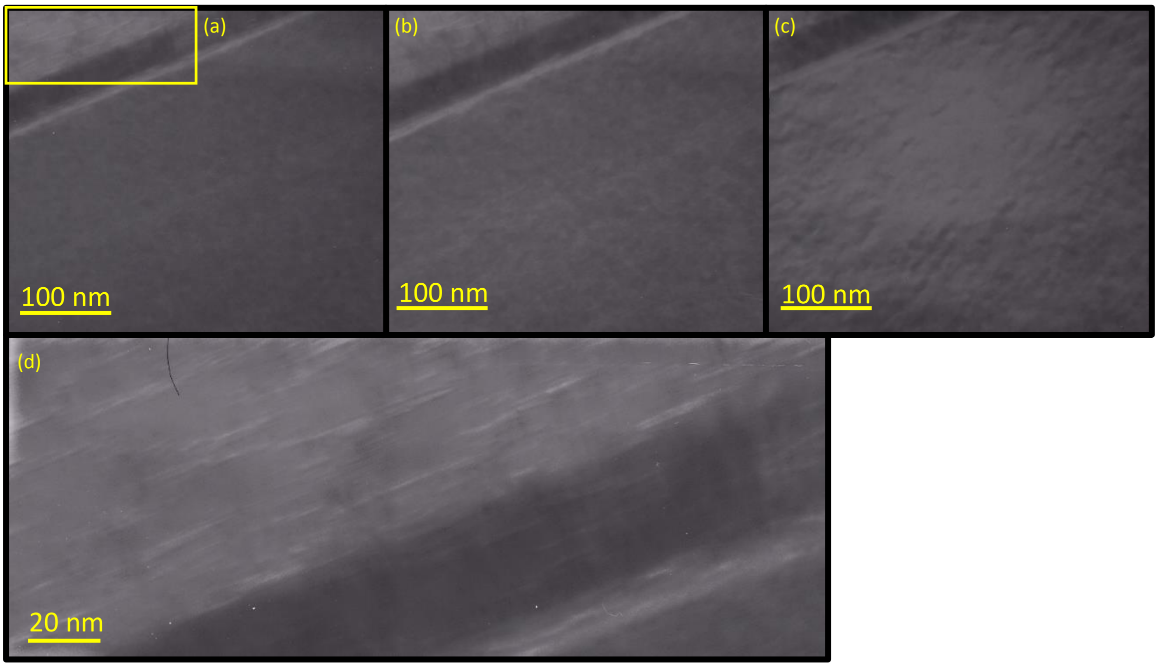

Supplementary figure 6. Sequential inverted colour TEM images of the same region of sample POG 3 (FIB) taken after (**a**) no previous exposure (**b**) 8 minutes exposure and (**c**) having been viewed at higher magnification for a further 7 minutes. Delaminations are present in the upper left part of the image even after no previous beam exposure (**d**). These delaminations do not appear to grow or form with increased exposure to the beam. Extensive beam exposure does however result in amorphisation, increased mottling and a loss of contrast.

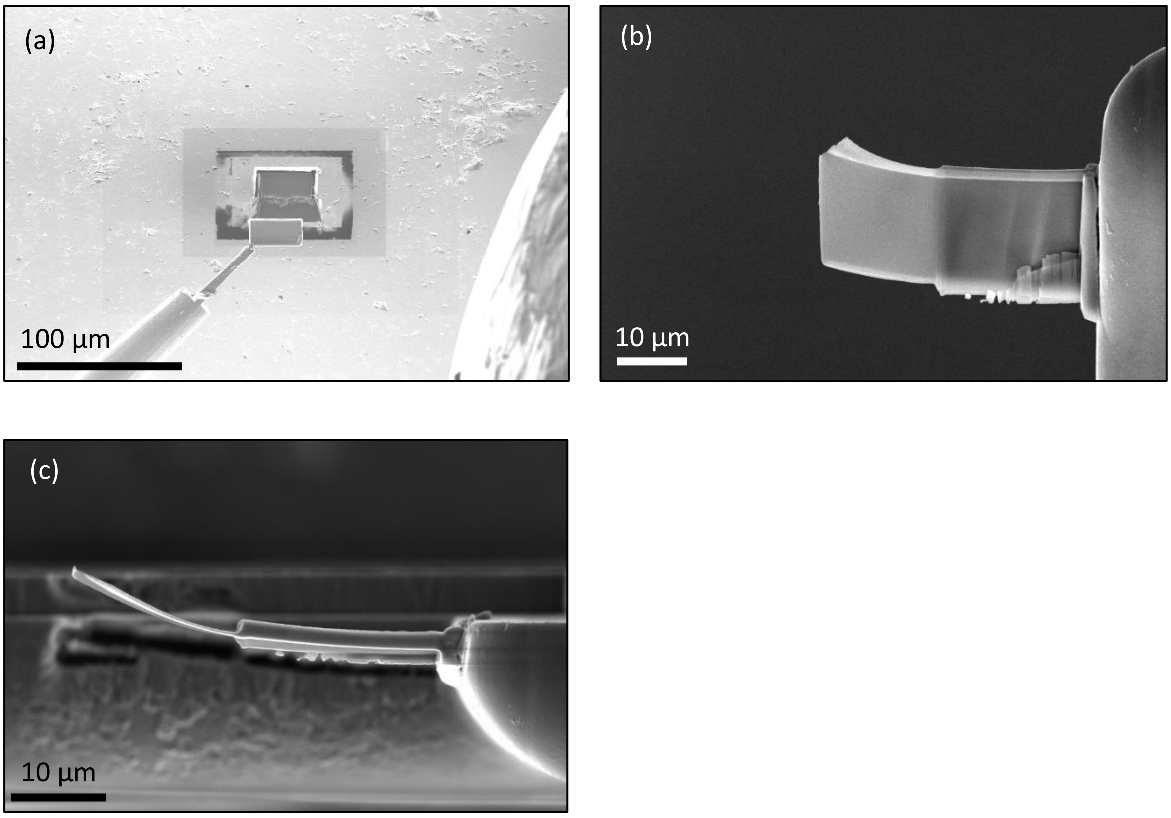

Supplementary figure 7. (**a**) Ion beam image of a sample slice being removed from the trenched out area by means of a micromanipulator. (**b**) Secondary electron image of the sample film after being attached to the Cu sample holder. The left-hand side has been thinned by the ion beam and shows extensive bending indicative of a release of stored elastic strain. (**c**) Edge on view of the sample as in (**b**).

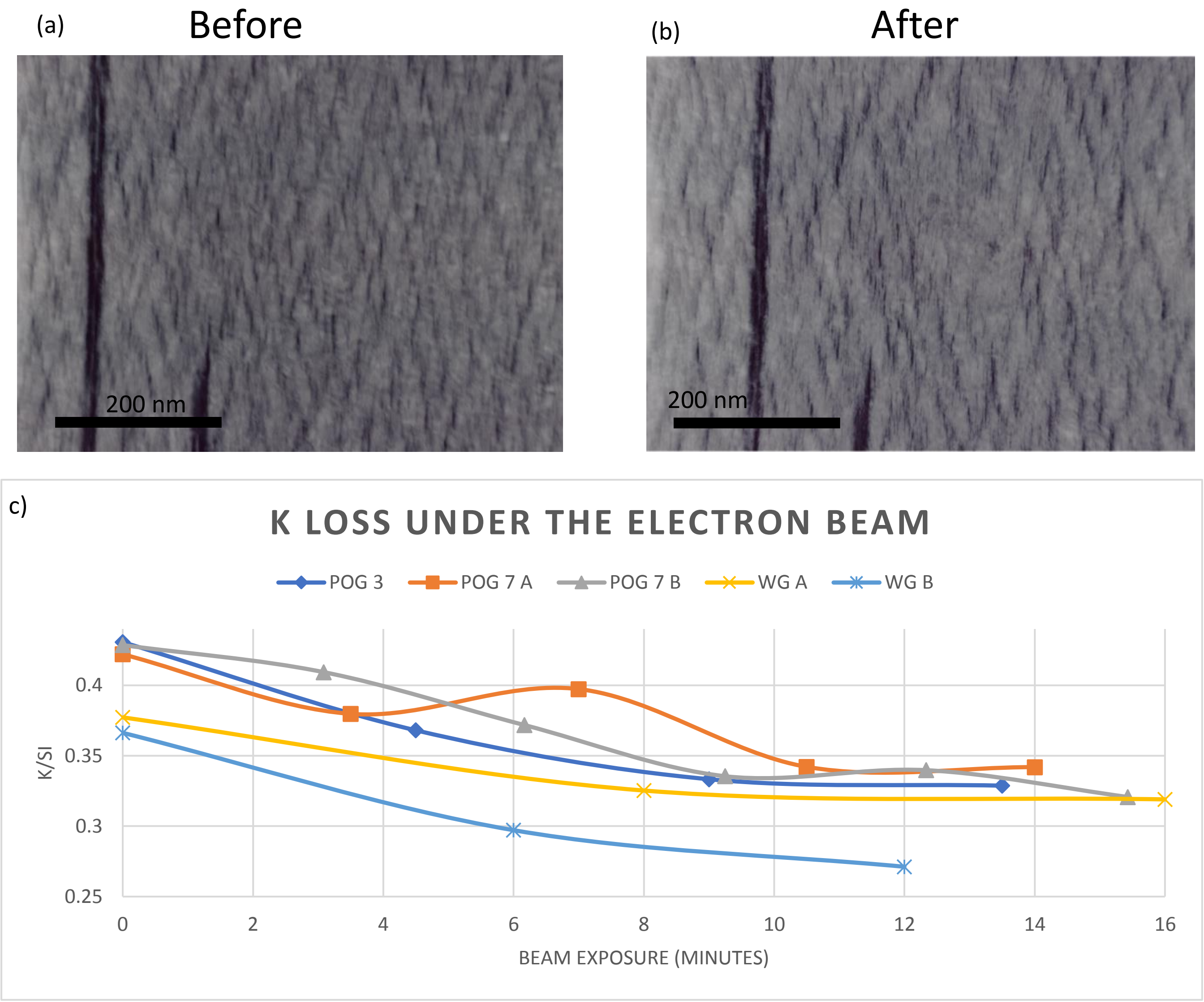

Supplementary Figure 8. (**a**) and (**b**) TEM micrographs of a region of biotite from sample POG 7 from which EDX spectra were collected. En-echelon arrays of delaminations were present both before and after EDX analysis using a focussed beam. Beam effects are visible and highlighted in (**b**) as amorphisation and removal of material around the beam spot. (**c**) Plot of K/Si ratio v time spent exposed to the beam as recorded in biotite from two different samples, POG 3, an ultramylonite and POG 7, a protomylonite. K is lost but the rate of decrease reduces over time and considerable K is retained in the biotite even after 15 minutes or more exposure. This is in contrast to the rapid and near total loss of Na previously reported in paragonite over a shorter timescale[28].

POG 3 FIB

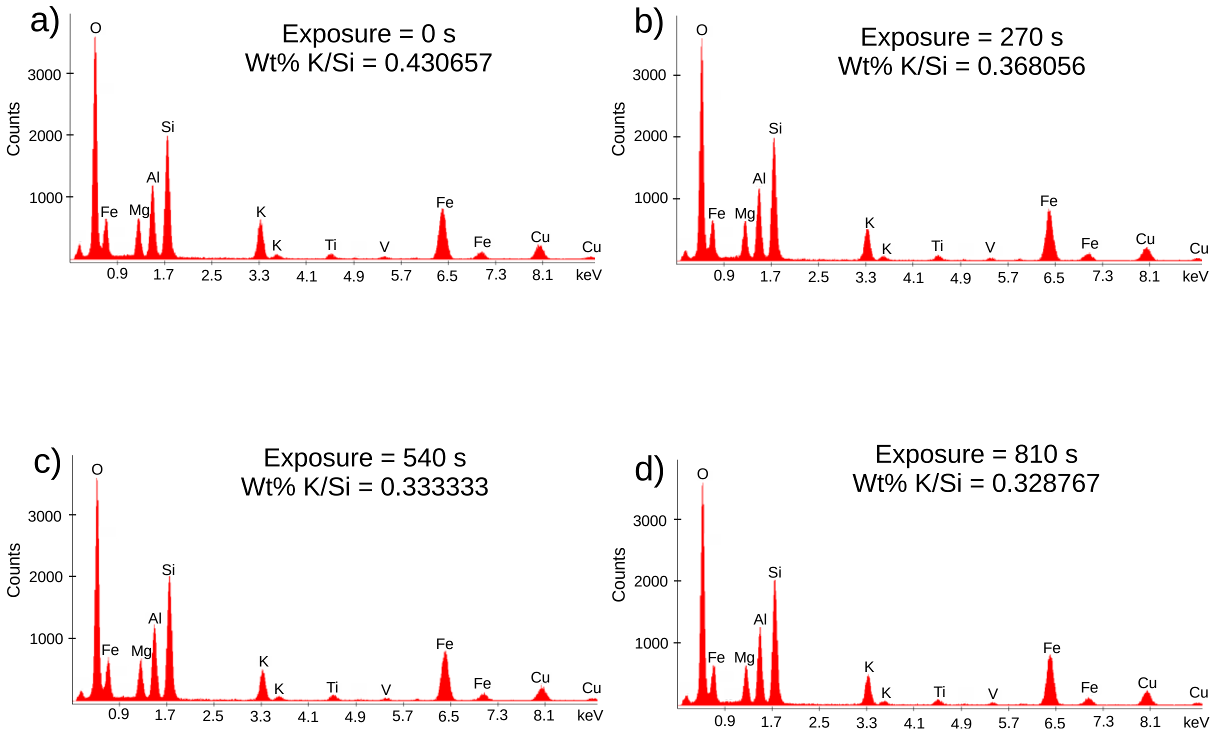

Supplementary figure 9. EDX spectra acquired from a biotite in sample POG 3, an ultramylonite. The exposure value indicates the amount of time the point had been exposed to the beam prior to beginning the acquisition, this increases from 0 s in (**a**) to 270 s in (**b**), 540 s in (**c**) and 810 s in (**d**). A small decrease in the height of the K peak at approximately 3.3 keV occurs over time. Acquisition time was 90 live seconds per spectrum,

POG 7 (grain A)

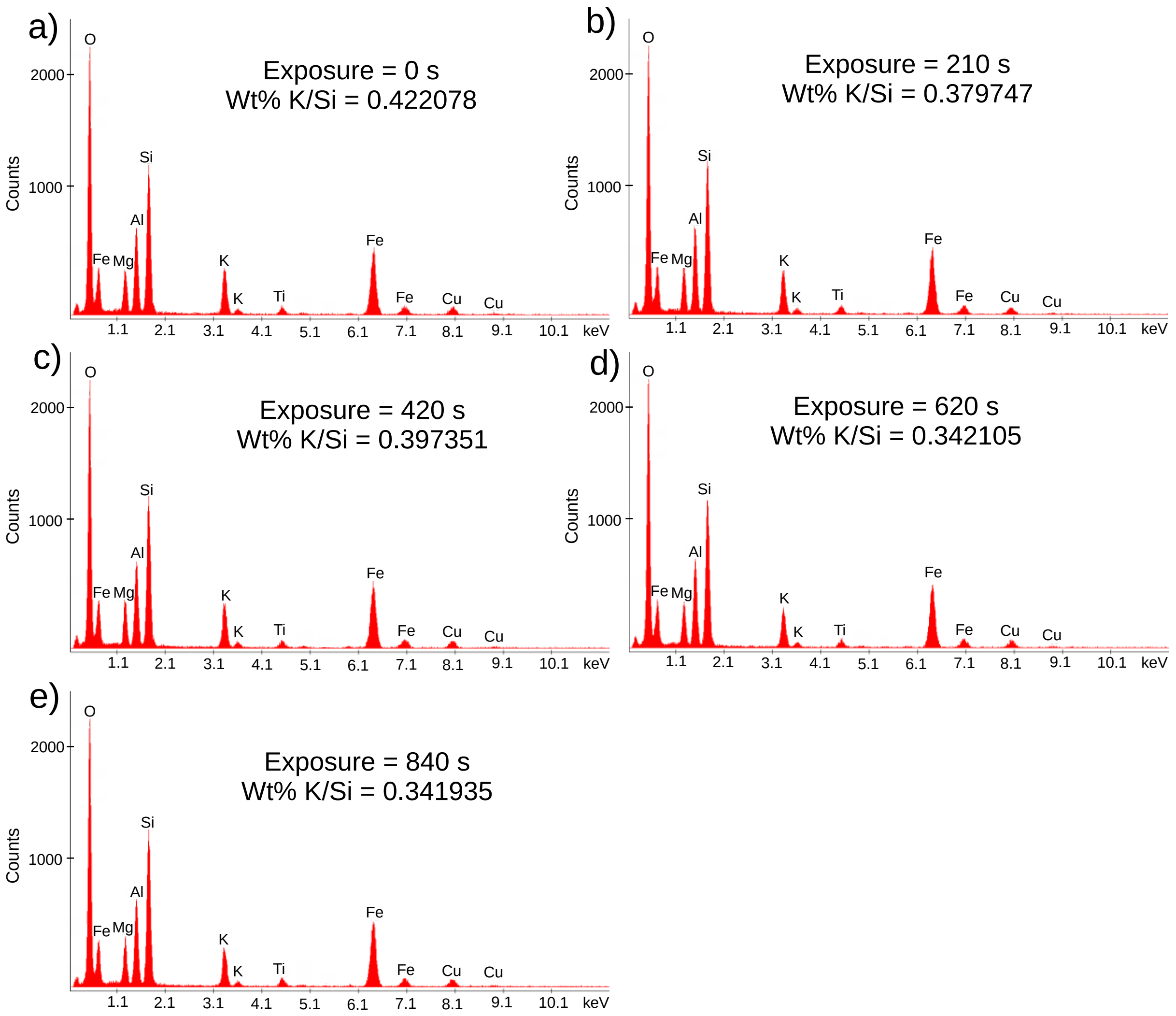

Supplementary figure 10. EDX Spectra acquired from a biotite in sample POG 7, a Protomylonite. This is the region that is imaged in figure 3 (a) and (b) before and after these spectra were collected. The exposure value indicates the amount of time the point had been exposed to the beam prior to beginning the acquisition (in this case the first spectra was collected after the site had been exposed to the beam briefly for around 15 seconds in order to collect the image used in supplementary figure 6a). The time increases from 0 s in (**a**) to 210 s in (**b**), 420 s in (**c**), 620 s in (**d**) and 840 s in (**e**). A small decrease in the height of the K peak at approximately 3.3 keV occurs over time. Acquisition time was 60 live seconds per spectrum, accelerating voltage used was 200 keV.

POG 7 (grain B)

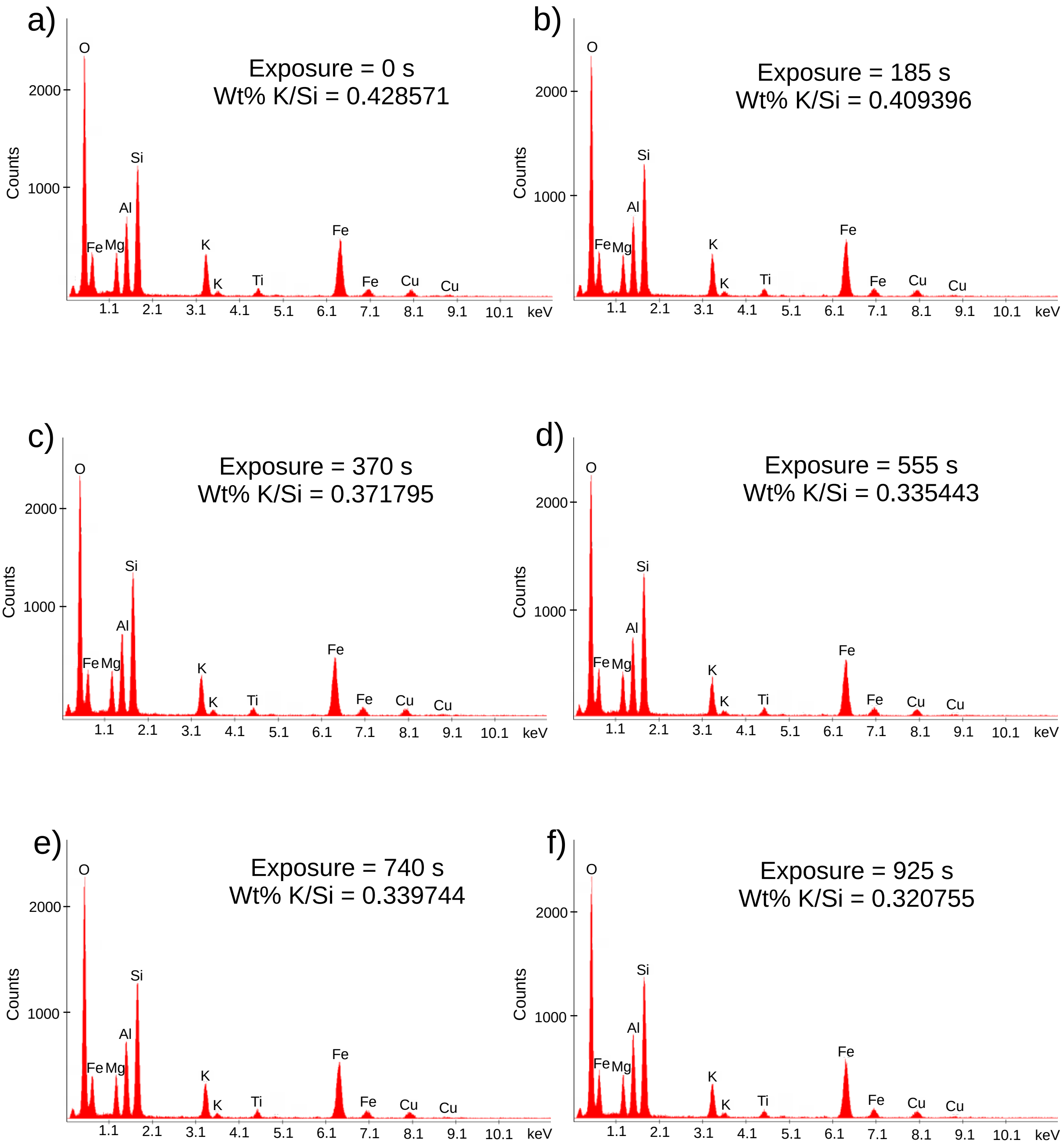

Supplementary figure 11. EDX Spectra acquired from a biotite in sample POG 7, a Protomylonite. The exposure value indicates the amount of time the point had been exposed to the beam prior to beginning the acquisition, this increases from 0 s in (**a**) to 185 s in (**b**), 370 s in (**c**), 555 s in (**d**), 740 s in (**e**) and 925 s in (**f**). A small decrease in the height of the K peak at approximately 3.3 keV occurs over time. Acquisition time was 60 live seconds per spectrum, accelerating voltage used was 200 keV.

Westerly granite (grain A)

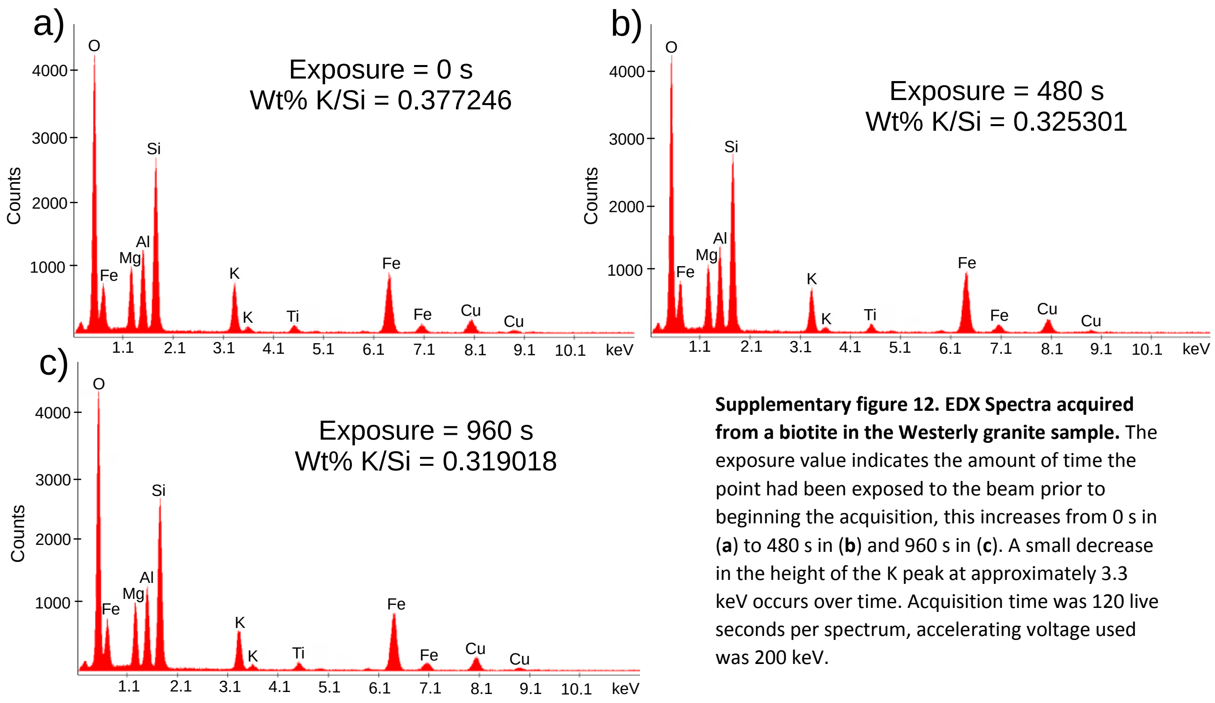

Supplementary figure 12. EDX Spectra acquired from a biotite in the Westerly granite sample. The exposure value indicates the amount of time the point had been exposed to the beam prior to beginning the acquisition, this increases from 0 s in (**a**) to 480 s in (**b**) and 960 s in (**c**). A small decrease in the height of the K peak at approximately 3.3 keV occurs over time. Acquisition time was 120 live seconds per spectrum, accelerating voltage used was 200 keV.

Westerly granite (grain B)

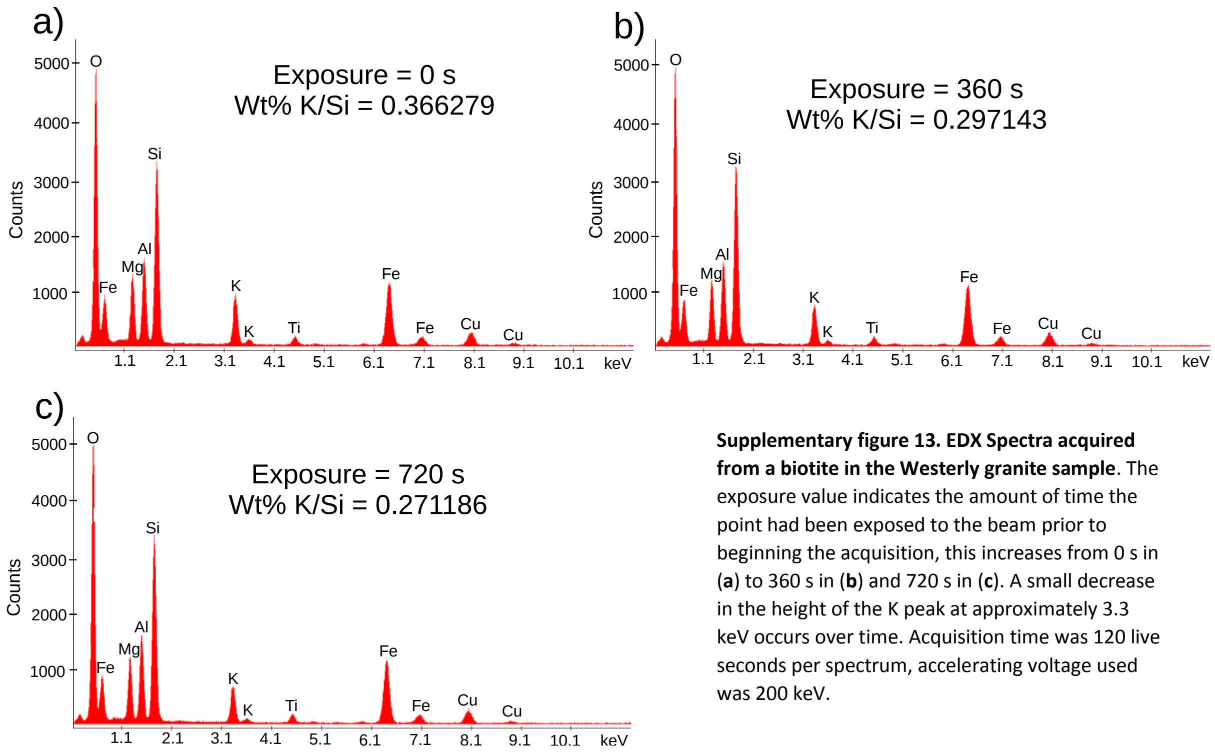

Supplementary figure 13. EDX Spectra acquired from a biotite in the Westerly granite sample. The exposure value indicates the amount of time the point had been exposed to the beam prior to beginning the acquisition, this increases from 0 s in (**a**) to 360 s in (**b**) and 720 s in (**c**). A small decrease in the height of the K peak at approximately 3.3 keV occurs over time. Acquisition time was 120 live seconds per spectrum, accelerating voltage used was 200 keV.

Table of samples

Sample	TEM foil name	Location	Lithology	Location in sample	Preparation	Instrument (accelerating voltage)	TEM mode	EDX performed
CMB5A	CMB5A 1	CMB line (Mte. Cerano)	Orthogneiss mylonite	Recrystallized halo of coarse biotite	FIB	2100 (200KeV)	Scanning	No
CMB5A	CMB5A 2	CMB line (Mte. Cerano)	Orthogneiss mylonite	Centre of coarse biotite	FIB	2100 (200KeV)	Scanning	No
CMB5A	CMB5A 3	CMB line (Mte. Cerano)	Orthogneiss mylonite	Matrix	FIB	2100 (200KeV)	Scanning	No
POG 3	POG 3 FIB	Pogallo line (Val Pogallo)	Orthogneiss ultramylonite	Matrix	FIB	2000FX (200KeV)	Imaging	Yes
POG 3	POG 3 PIPS	Pogallo line (Val Pogallo)	Orthogneiss ultramylonite	Matrix	PIPS	2000FX (200KeV)	Imaging	No
POG 7	POG 7 FIB	Pogallo line (Val Pogallo)	Orthogneiss protomylonite	Matrix biotite grain	FIB	2000FX (200KeV)	Imaging	Yes (x2)
POG 7	POG 7 PIPS	Pogallo line (Val Pogallo)	Orthogneiss protomylonite	Matrix	PIPS	2000FX (200KeV)	Imaging	No
WG	WG FIB	Rhode island, USA	Granite	Coarse biotite grain	FIB	2000FX (200KeV)	Imaging	Yes (x2)
WG	WG PIPS	Rhode island, USA	Granite	Groundmass biotite grain	PIPS	2000FX (200KeV)	Imaging	No

Supplementary table 1: List of samples and TEM foils used. Within the instrument column, 2100 refers to a CEOS GmbH "CESCOR" probe side aberration corrected JEOL 2100FCs TEM while 200FX refers to a tungsten filament JEOL 2000FX TEM.

Supplementary references

1. Etheridge, M. A., Hobbs, B. E. & Paterson, M. S. Experiental deformation of single crystals of biotite. *Contrib. to Mineral. Petrol.* **38,** 21–36 (1973).
2. Bell, I. A., Wilson, C. J. L., McLaren, A. C. & Etheridge, M. A. Kinks in mica: Role of dislocations and (001) cleavage. *Tectonophysics* **127,** 49–65 (1986).
3. Behrmann, J. H. A study of white mica microstructure and microchemistry in a low grade mylonite. *J. Struct. Geol.* **6,** 283–292 (1984).
4. Kushima, A., Qian, X., Zhao, P., Zhang, S. & Li, J. Ripplocations in van der Waals layers. *Nano Lett.* **15,** 1302–1308 (2015).
5. Gruber, J. *et al.* Evidence for Bulk Ripplocations in Layered Solids. *Sci. Rep.* **6,** 1–8 (2016).
6. Siegesmund, S. *et al.* Exhumation and deformation history of the lower crustal section of the Valstrona di Omegna in the Ivrea Zone, southern Alps. *Tecton. Asp. Alpine-Dinaride-Carpathian Syst.* **298,** 45–68 (2008).
7. Boriani, a., Burlini, L. & Sacchi, R. The Cossato-Mergozzo-Brissago Line and the Pogallo Line (Southern Alps, Northern Italy) and their relationships with the late-Hercynian magmatic and metamorphic events. *Tectonophysics* **182,** 91–102 (1990).

8. Meike, A. In situ deformation of micas: a high-voltage electron-microscope study. *Am. Mineral.* **74,** 780–796 (1989).

9. Mariani, E., Brodie, K. H. & Rutter, E. H. Experimental deformation of muscovite shear zones at high temperatures under hydrothermal conditions and the strength of phyllosilicate-bearing faults in nature. *J. Struct. Geol.* **28,** 1569–1587 (2006).

10. Mariani, E. An Experimental Study of the Deformation of White Mica. *PhD Thesis, University of Manchester* (2002).
